# Dual Substitution and Spark Plasma Sintering to Improve Ionic Conductivity of Garnet Li_7_La_3_Zr_2_O_12_

**DOI:** 10.3390/nano9050721

**Published:** 2019-05-10

**Authors:** Zhencai Dong, Chao Xu, Yongmin Wu, Weiping Tang, Shufeng Song, Jianyao Yao, Zhengyong Huang, Zhaoyin Wen, Li Lu, Ning Hu

**Affiliations:** 1 College of Aerospace Engineering, Chongqing University, Chongqing 400044, China; 20163113037@cqu.edu.cn; 2 College of Electrical Engineering, Chongqing University, Chongqing 400044, China; xuchao1995@cqu.edu.cn (C.X.); huangzhengyong@cqu.edu.cn (Z.H.); 3 State Key Laboratory of Space Power-sources Technology, Shanghai Institute of Space Power-Sources, Shanghai 200245, China; wuym2014@126.com (Y.W.); tangwp@sina.cn (W.T.); 4 CAS Key Laboratory of Materials for Energy Conversion, Shanghai Institute of Ceramics, Chinese Academy of Sciences, Shanghai 200050, China; zywen@mail.sic.ac.cn; 5 Department of Mechanical Engineering, National University of Singapore, Singapore 117575, Singapore; luli@nus.edu.sg; 6 National University of Singapore Suzhou Research Institute, Suzhou 215024, China; 7 School of Mechanical Engineering, Hebei University of Technology, Tianjin 300401, China; 8 State Key Laboratory of Coal Mine Disaster Dynamics and Control, Chongqing University, Chongqing 400044, China

**Keywords:** garnet, dual substitution, spark plasma sintering, conductivity

## Abstract

Garnet Li_7_La_3_Zr_2_O_12_ is one of the most promising solid electrolytes used for solid-state lithium batteries. However, low ionic conductivity impedes its application. Herein, we report Ta-doping garnets with compositions of Li_7-x_La_3_Zr_2-x_Ta_x_O_12_ (0.1 ≤ x ≤ 0.75) obtained by solid-state reaction and free sintering, which was facilitated by graphene oxide (GO). Furthermore, to optimize Li_6.6_La_3_Zr_1.6_Ta_0.4_O_12_, Mg^2+^ was select as a second dopant. The dual substitution of Ta^5+^ for Zr^4+^ and Mg^2+^ for Li^+^ with a composition of Li_6.5_Mg_0.05_La_3_Zr_1.6_Ta_0.4_O_12_ showed an enhanced total ionic conductivity of 6.1 × 10^−4^ S cm^−1^ at room temperature. Additionally, spark plasma sintering (SPS) was applied to further densify the garnets and enhance their ionic conductivities. Both SPS specimens present higher conductivities than those produced by the conventional free sintering. At room temperature, the highest ionic conductivity of Li_6.5_Mg_0.05_La_3_Zr_1.6_Ta_0.4_O_12_ sintered at 1000 °C is 8.8 × 10^−4^ S cm^−1^, and that of Li_6.6_La_3_Zr_1.6_Ta_0.4_O_12_ sintered at 1050 °C is 1.18 × 10^−3^ S cm^−1^.

## 1. Introduction

Nowadays, commercial lithium ion batteries generally employ organic liquid electrolytes, which have caused a series of problems and hindered development of the batteries. The formation of lithium dendrites during cycling lurks between the solid-liquid interface because of the uneven distribution of current and lithium ions, causing safety issues. Moreover, the liquid electrolytes are flammable, exacerbating these safe issues. Therefore, the replacement of liquid electrolytes with solid ones for lithium batteries is of great significance [1,2]. All-solid-state lithium batteries have been widely studied for electric vehicles, rail transit, energy storage and aerospace fields, because of their promising energy density, long cycling life and excellent safety. As one of the most promising solid electrolytes, the lithium-rich garnet-type compound with a nominal composition of Li_7_La_3_Zr_2_O_12_ (LLZO) has many advantages, especially in Li-ion conductivity [3,4]. It has been shown that LLZO exists in two phases, namely a tetragonal phase with an inferior Li-ion conductivity of about 10^−6^ S cm^−1^ at room temperature, and a cubic phase with a superior Li-ion conductivity of about 10^−4^ S cm^−1^ at room temperature. In order to achieve higher conductivities, the content of Li is usually decreased below 7 Li per formula unit, while the concentration of Li vacancies is increased in LLZO through substitution. It was reported that the replacement of Zr^4+^ with Ta^5+^ in LLZO is an effective route to increase the concentration of Li vacancies and to stabilize the cubic garnet [5]. The other effective solutions to reduce the Li content are through adding dopants like Al^3+^, Ga^3+^, etc. [6,7]. The principle is occupying the Li sites directly as the same as the substitution at the Zr sites. Among the dopants, Mg^2+^ has been reported as a sintering aid which can lower the sintering temperature and improve the relative density. Mg^2+^ serves as a sintering aid with a mechanism of grain-boundary diffusion and simultaneously suppresses the recrystallization [8]. The most common method for preparing garnet-type solid electrolytes is through the solid state method (SSR) and free sintering, which is widely applied in industry due to its low cost. However, under the condition of a high sintering temperature (>1200 °C) and a long sintering time (>16 h), it is hard to obtain dense ceramic pellets (relative density > 90%) by free sintering. As a result, various advanced sintering technologies have been developed to enhance the relative density, such as hot pressing, flowing oxygen sintering, field-assisted sintering and spark plasma sintering (SPS). It has been reported that the sintering theory of SPS, also famous as field assisted sintering technique (FAST) and pulsed electric current sintering (PECS), is different from the conventional sintering technique. In the sintering process, the DC current flowing through the graphite die generates huge local joule heat. Combining with the uniaxial high pressure, the contact area between particles starts to fuse, resulting in rapid densification [9,10]. This principle can effectively improve the relative density and lower the sintering temperature. Besides, it greatly shortens the sintering time and simplifies the process because the sinter does not need the mother powders to compensate the loss of lithium in the vacuum environment [11].

In this work, we investigated a Ta-doped garnet composed of Li_7-x_La_3_Zr_2-x_Ta_x_O_12_ (x = 0.75, 0.6, 0.5, 0.4, 0.3, 0.2, 0.1) (LLZTO) through the traditional method. The preparation process was facilitated by graphene oxide (GO) and was divided into two procedures. Firstly, the raw material was calcined to a cubic precursor at 900 °C with 1 wt.% GO with respect to the total mass of the starting materials. The cubic precursor was then sintered at a relatively high temperature. By using GO as a sacrificial template, a nanoscale garnet powder with superior sinterability was produced [12,13]. Furthermore, the LLZTO composition was optimized, and Mg was served as the second dopant to further increase the conductivity. In order to compare with the conventional free sintering method and to further improve the ionic conductivity, the optimized Ta-doping and Ta/Mg dual substituted samples were sintered via spark plasma sintering.

## 2. Experimental

### 2.1. Synthesis and Free Sintering

LLZTO powders were synthesized by a traditional solid-state method. The primary materials include LiNO_3_ (≥99.9%, Shanghai Aladdin Biochemical Technology Co. Ltd, Shanghai, China), MgO (98.0%, Shanghai Aladdin Biochemical Technology Co. Ltd, Shanghai, China), La_2_O_3_ (99.9%, Shanghai Aladdin Biochemical Technology Co. Ltd, Shanghai, China), ZrO_2_ (99.99%, Shanghai Aladdin Biochemical Technology Co. Ltd, Shanghai, China), and Ta_2_O_5_ (99.5%, Shanghai Aladdin Biochemical Technology Co. Ltd, Shanghai, China). During the sintering process, a portion of the lithium salt is decomposed into Li_2_O and lost at high temperature [14]. To compensate for the lithium loss, an excess of LiNO_3_ is added. Actually, we tried different amounts of LiNO_3_ excess and found that the 40 wt.% excess was better. It was reported that the excess Li_2_O can densify the lithium garnet by forming glass-like compound at the grain-boundaries, and thus improve the ionic conductivity [15]. La_2_O_3_ was dried at 900 °C for 12 h to remove the moisture. A total amount of 1 wt.% GO with respect to the total mass of the starting materials was added to inhibit the aggregation and to obtain the nano-grains of garnet powders. The preparation of GO adopted an improved Hummers method [16,17,18,19]. Firstly, a 10:1 mixture of concentrated H_2_SO_4_/H_3_PO_4_ (250:25 mL) was added to 2 g graphite flakes (XFNAN, 99.5 wt.%, 500 μm) and a slight exotherm was observed. Then, the mixed solution was placed in an ice bath, and 12 g KMnO_4_ was slowly added. The solution was well-stirred after being transferred to a magnetic mixer with a constant temperature of 50 °C. After cooling to room temperature, the mixture of 9.56 mL 30% H_2_O_2_ and 134 mL deionized water was added to the above solution to end the oxidation reaction. To wipe off impurities such as K, P, and Mn, it is necessary to control the purification process. The raw GO solution was filtered to remove solid impurities and dialyzed through a semi-permeable membrane to remove soluble impurities. Subsequently, the solution was separated into centrifuge tubes and washed by deionized water and dilute hydrochloric acid alternately until the solution approaching to a neutral pH. Finally, the GO solution with a concentration of 3.1 mg/mL was obtained.

The stoichiometric materials with the GO solution were mixed and ball-milled for 4 h in 3-Methyl-1-butanol (98.5%, Aladdin) with a planetary ball-mill machinery, in which zirconia balls and a jar were used. The milling powder was dried in an oven for 3 h at 90 °C and pressed into cylindrical pellets. The pellets were placed on a MgO ceramic wafer in an Al_2_O_3_ crucible so as to isolate from other elements contaminations. After calcined at 900 °C for 3 h in air, the pellets were ground to LLZTO powders, and the second ball-milling process was repeated. Then, the LLZTO powders were dried and pressed into cylindrical pellets. Similarly, the samples were covered with corresponding mother powers to reduce lithium loss and sintered at 1100 °C for 12 h. The obtained ceramic pellets were polished using metallographic abrasive papers with grit numbers of 180, 600 and 1200, respectively. The chemical reactions occurring during the synthesis of LLZTO are as follows:

4(7-x)LiNO_3_ + 6La_2_O_3_ + 4(2-x)ZrO_2_ + 2xTa_2_O_5_ = 4Li_7-x_La_3_Zr_2-x_Ta_x_O_12_ + 4(7-x)NO_2_↑ + (7-x)O_2_↑ (1)

260LiNO_3_ + 2MgO + 60La_2_O_3_ + 64ZrO_2_ + 8Ta_2_O_5_ = 40Li_6.5_Mg_0.05_La_3_Zr_1.6_Ta_0.4_O_12_ + 260NO_2_↑ + 65O_2_↑ (2)

### 2.2. SPS Sintering

The optimized Ta doping and Mg/Ta dual substituted garnets were further sintered by SPS at different temperatures (950 °C, 1000 °C and 1050 °C) for 10 min. The SPS procedure was conducted on a SPS machine (SPS-211Lx, Fuji Radio Engineering Machinery Co., Ltd, Nagoya-shi, Japan) using a graphite mold (30 mm in height × 10.5 mm in diameter) with two corresponding punches (10 mm in diameter). Prior to the SPS process, the above-mentioned ceramic chips after testing were re-ground and sieved to obtain uniform fine particles so that the powders could have a better sintering during the SPS procedure. In order to demold easily, the inserted sheets were used to separate the powders and the graphite mold. The SPS was conducted in the vacuum chamber and the heating rate was set to 50 °C min^−1^. When the temperature was raised to the target sintering temperature, a static uniaxial pressure of 50 MPa was applied to the samples. After holding for 10 min, the specimens in the chamber were cooled naturally to 600 °C with pressure decreasing to 5 MPa, which contributes the densification of the samples during shrinking. Then, the pressure was removed to avoid the formation of cracks as the temperature naturally cooled down to room temperature. All the procedures were implemented through the setup program controlled under the automatic operation mode. The sintered samples obtained by SPS were polished by the same way as that for previous sintered pellets.

### 2.3. Characterization

The relative density was calculated using the following equation:

(3) $$d=\frac{m}{\rho \pi r^{2}l}\times 100\%$$

(4) $$\rho =\frac{MZ}{N_{A}V}$$

where, *m* represents the mass, *r* represents the radius, and *l* represents the height of the cylinders. The relative density is the ratio of the measured density at room temperature to the theoretical density ρ. *M*, *Z* and *N_A_* represent the molecular mass, number of molecular per unit cell, and Avogadro’s constant, respectively. *V* represents the cell volume which is decided by the lattice parameter. The final sample is a cylinder-shaped product. The phases of the powders calcined at 900 °C and 1100 °C were examined by X-ray diffraction (XRD, D2 PHASER, Bruker, Beijing, China) in the 2θ range of 10° and 80°. The X-ray data were collected with a step of 0.01° and a counting time of 0.3 s per step. The cross-sectional morphologies of the pellets were revealed by scanning electron microscopy (SEM, Phenom ProX, Eindhoven, The Netherlands) with an acceleration voltage of 15 kV. Electrochemical impedance spectroscopy (EIS) measurements were conducted on the polished pellets. Both surfaces of the pellet were coated with a silver paste as block electrodes. Since the garnet-type electrolyte is prone to react with moisture and carbon dioxide in the air, all ceramic pellets were encapsulated in Swagelok cells in a glove box filled with inert gas. The samples were measured in the temperature range of room temperature and 130 °C. An amplitude of 10 mV and a frequency of 1 MHz–0.1 Hz were set on an Autolab PGSTAT302N System. The ionic conductivities were then calculated based on the following equation:

(5) $$\sigma =\frac{l}{\pi r^{2}R}$$

where, *l* represents the height of the cylinders, *r* represents the radius and *R* represents the resistance obtained from the impedance chart. Furthermore, the activation energy *E_a_*, representing the migration barrier of ions, was calculated by the Arrhenius equation:

(6) $$\sigma T=\mathrm{Aexp}\left(-E_{a}/\mathrm{kT}\right)$$

where, A, k, and T represent the pre-exponential parameter, Boltzmann constant, and thermodynamic temperature, respectively.

## 3. Results and Discussion

**Figure 1:**
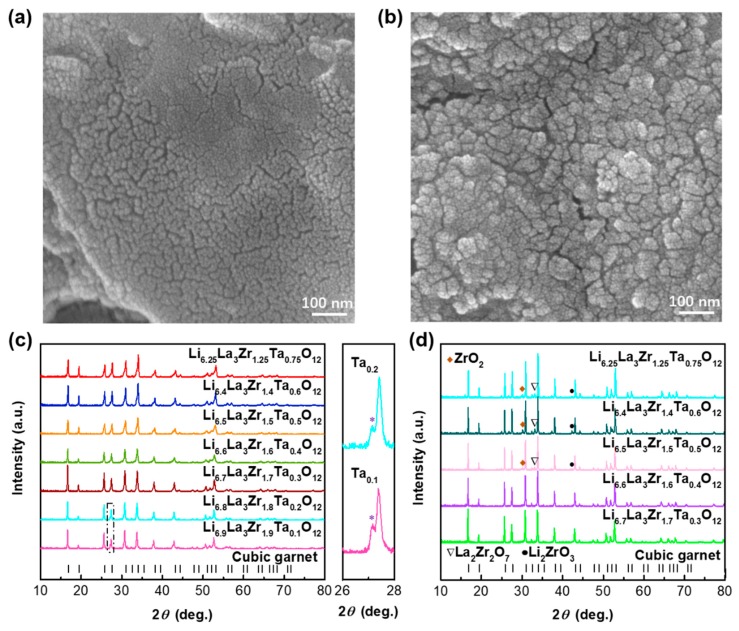
SEM of LLZTO calcined at 900 °C with GO (**a**) and without GO (**b**). The XRD results of the Ta-doping and Ta/Mg-dual substituted garnet calcined at 900 °C (**c**) and sintered at 1100 °C (**d**). The black vertical lines at the bottom represent the reference cubic garnet (PDF#45-0109).

LLZTO powder was synthesized by solid-state reaction with GO as the sacrificial template. As seen in Figure 1a,b, the grain size of the LLZTO powder is about 10–20 nm in the presence of GO, whereas, the grains are 20–30 nm in size and irregular morphology in the absence of GO. It is indicated that the regular and fine grains facilitated sinter, though the aggregation was not completely prevented by GO. The XRD patterns of the Ta-doping garnets with compositions of Li_7-x_La_3_Zr_2-x_Ta_x_O_12_ (x = 0.75, 0.6, 0.5, 0.4, 0.3, 0.2, 0.1) calcined at 900 °C and sintered at 1100 °C are shown in Figure 1c,d. As seen in Figure 1c, all the components formed the cubic phase after calcined at 900 °C except in the range of x ≤ 0.2, where the mixed cubic and tetragonal phases were found.

It should be noted that the garnets with compositions of Li_6.9_La_3_Zr_1.9_Ta_0.1_O_12_ and Li_6.8_La_3_Zr_1.8_Ta_0.2_O_12_ were not well-pelleted after sintering at 1100 °C. Therefore, the XRD patterns of Li_6.9_La_3_Zr_1.9_Ta_0.1_O_12_ and Li_6.8_La_3_Zr_1.8_Ta_0.2_O_12_ are not presented in Figure 1d. As seen in Figure 1d, the garnets with compositions of Li_6.7_La_3_Zr_1.7_Ta_0.3_O_12_ and Li_6.6_La_3_Zr_1.6_Ta_0.4_O_12_ showed the single cubic garnet phase, whereas the garnets with the Ta content of above 0.4 contained some impurities. The single cubic phase indicates fast ion conduction while the impurities hinder ion conduction. The following EIS results also demonstrated the influence of Ta substitution on ionic conductivity.

**Figure 2:**
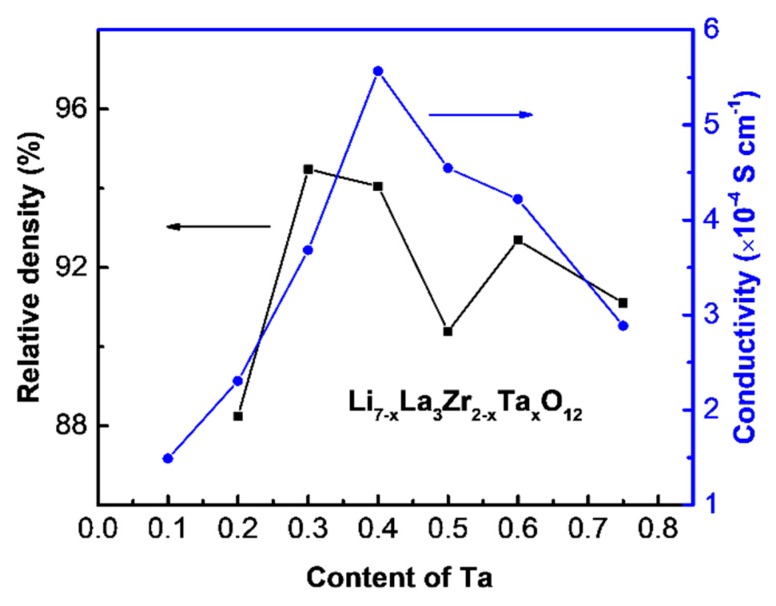
Relative density and ionic conductivity of LLZTO sintering pellets of various components.

The relative densities and ionic conductivities of Li_7-x_La_3_Zr_2-x_Ta_x_O_12_ (x = 0.75, 0.6, 0.5, 0.4, 0.3, 0.2, 0.1) sintering by traditional methods are shown in Figure 2. The growing and following declining trend is for relative density to vary with addition of Ta. It is indicated that the enhancement of sinterability might have a significant impact on conductivities. Not unexpectedly, the ionic conductivity showed a noticeable variation. Obviously, it is increased firstly and then decreased with the substitution of Ta for Zr. Li_6.6_La_3_Zr_1.6_Ta_0.4_O_12_ possessed the highest conductivity of about 5.6 × 10^−4^ S cm^−1^. The increase in relative density is highly beneficial to ion conduction. However, it seems that the variation of ionic conductivity is not always consistent with that of relative density, which proves that relative density is not the unique factor affecting ion conductivity. Besides, the ion conduction was also determined by the crystal structure and the Li content. An excess substitution of Ta for Zr led to impurities and a low Li content, which in turn decreased the ionic conductivity.

The refined lattice parameters of LLZTO have been reported by some groups [20,21,22,23,24]. It can be seen from Table 1 that the lattice parameter of LLZTO increases with the increasing content of Ta. Based on the lattice parameters, the theoretical densities and corresponding relative densities are calculated by Equations (3) and (4).

**Figure 3:**
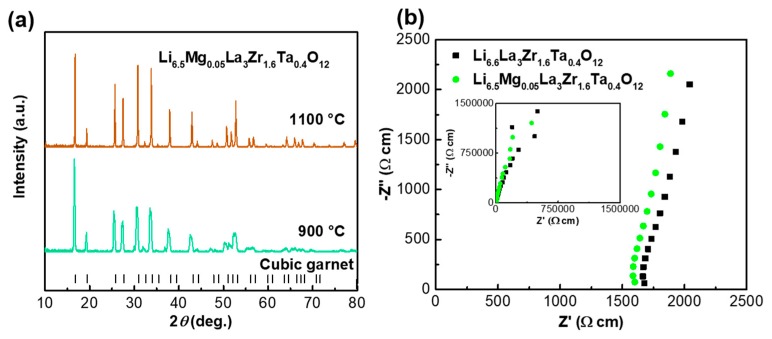
(**a**) XRD of Li_6.5_Mg_0.05_La_3_Zr_1.6_Ta_0.4_O_12_ treated at 900 °C and 1100 °C. (**b**) Nyquist plots of Li_6.6_La_3_Zr_1.6_Ta_0.4_O_12_ and Li_6.5_Mg_0.05_La_3_Zr_1.6_Ta_0.4_O_12_ sintered by free sintering; the insets are impedance plots (10 Hz–1 MHz) measured in air at room temperature.

We subsequently designed a dual substitution of Mg for Li and Ta for Zr with a composition of Li_6.5_Mg_0.05_La_3_Zr_1.6_Ta_0.4_O_12_ based on the optimal composition of Li_6.6_La_3_Zr_1.6_Ta_0.4_O_12_, because Mg is usually used as the sintering aid. As seen in Figure 3a, the XRD patterns show that Li_6.5_Mg_0.05_La_3_Zr_1.6_Ta_0.4_O_12_ exhibits a single cubic phase after calcined at 900 °C, which would be a beneficial effect on the final sinter. Li_6.5_Mg_0.05_La_3_Zr_1.6_Ta_0.4_O_12_ demonstrated a single cubic phase after sintered at 1100 °C. The cubic Li_6.5_Mg_0.05_La_3_Zr_1.6_Ta_0.4_O_12_ exhibited a relatively high conductivity of 6.1 × 10^−4^ S cm^−1^ compared with that of Li_6.6_La_3_Zr_1.6_Ta_0.4_O_12_ (Figure 3b). The improved ionic conductivity is probably because the dual substitution promotes the densification and adjusts the Li content.

**Figure 4:**
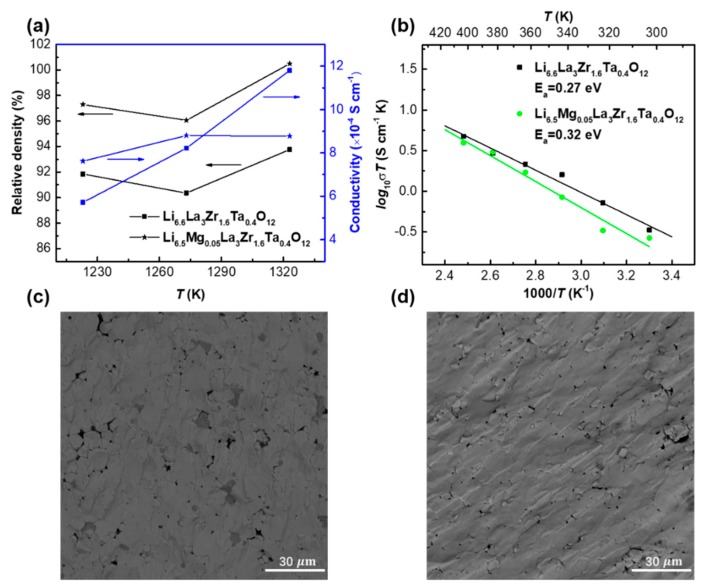
(**a**) Relative densities and ionic conductivities of Li_6.6_La_3_Zr_1.6_Ta_0.4_O_12_ and Li_6.5_Mg_0.05_La_3_Zr_1.6_Ta_0.4_O_12_ sintered by SPS at different temperatures. (**b**) Arrhenius plots of total ionic conductivities of Li_6.6_La_3_Zr_1.6_Ta_0.4_O_12_ and Li_6.5_Mg_0.05_La_3_Zr_1.6_Ta_0.4_O_12_ sintered by SPS. The dash points are experimental values and the solid lines are the fitting ones. The cross-section SEM of Li_6.6_La_3_Zr_1.6_Ta_0.4_O_12_ (**c**) and of Li_6.5_Mg_0.05_La_3_Zr_1.6_Ta_0.4_O_12_ (**d**) after conducting SPS at 1000 °C.

Figure 4a shows the relative densities and ionic conductivities of Li_6.6_La_3_Zr_1.6_Ta_0.4_O_12_ and Li_6.5_Mg_0.05_La_3_Zr_1.6_Ta_0.4_O_12_ sintered at different temperatures (950 °C, 1000 °C, and 1050 °C) by SPS. Since the Mg content is very low, the lattice parameter of Li_6.5_Mg_0.05_La_3_Zr_1.6_Ta_0.4_O_12_ should be similar with that of Li_6.6_La_3_Zr_1.6_Ta_0.4_O_12_ (12.939 Å [24]). For the relative density, both components showed a general increasing trend with the increase of SPS temperature, indicating that the increasing SPS temperature is in favour of densification. The room-temperature conductivity of the Ta-doping garnet Li_6.6_La_3_Zr_1.6_Ta_0.4_O_12_ was increased with the SPS temperature. Li_6.6_La_3_Zr_1.6_Ta_0.4_O_12_ possessed the highest total (bulk + grain boundary) ionic conductivity of 1.18 × 10^−3^ S cm^−1^ after treating at a SPS temperature of 1050 °C, while the Mg/Ta dual substituted Li_6.5_Mg_0.05_La_3_Zr_1.6_Ta_0.4_O_12_ exhibited a different variation. Li_6.5_Mg_0.05_La_3_Zr_1.6_Ta_0.4_O_12_ showed the highest total ionic conductivity of 8.8 × 10^−4^ S cm^−1^ after treating at a SPS temperature of 1000 °C. It indicated that the relatively high lithium content resulted in a relatively high ionic conductivity under a similar densification condition. Clearly, the SPS procedure enhanced the ionic conductivity compared with the traditional solid-state reaction. Figure 4b shows the dependence of the ionic conductivities on the temperatures for both Li_6.6_La_3_Zr_1.6_Ta_0.4_O_12_ and Li_6.5_Mg_0.05_La_3_Zr_1.6_Ta_0.4_O_12_ in the range between room temperature and 130 °C. It can be seen that both samples have a great thermal stability and the activation energies of Li_6.6_La_3_Zr_1.6_Ta_0.4_O_12_ and Li_6.5_Mg_0.05_La_3_Zr_1.6_Ta_0.4_O_12_ were calculated to be 0.27 eV and 0.32 eV, respectively.

The cross-section SEM micrographs of the specimens obtained from the SPS process are shown in Figure 4c,d. It can be seen that SPS specimens possessed a remarkable densification, same with the previous reports [9,10]. The fracture only occurred within the grain, not at the grain boundary, indicating the cohesive force between particles was increased, which strengthened the integration of the grain boundary. The increased density of the microstructure improved the ion conduction.

**Figure 5:**
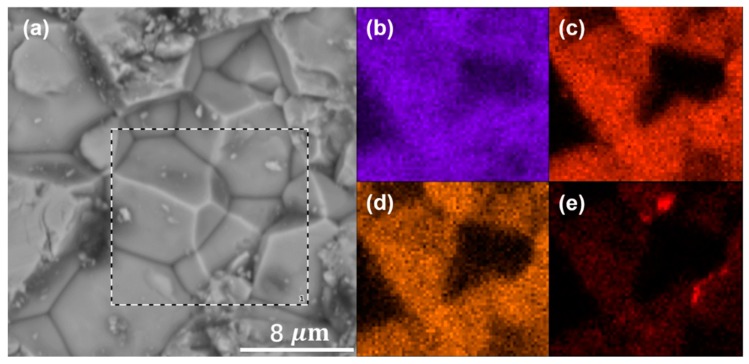
EDS mapping on the surface of Li_6.5_Mg_0.05_La_3_Zr_1.6_Ta_0.4_O_12_ sintered by SPS at 1050 °C: (**a**) mapping area, (**b**) La, (**c**) Zr, (**d**) Ta, and (**e**) Mg.

The uniform distribution of garnet elements is illustrated in the EDS mapping. As shown in Figure 5, elements La, Zr, Ta, and Mg were uniformly distributed in the grains. There is no obvious trace of Mg in grain-boundaries, which proved that most of element Mg is probably integrated to the lattice.

## 4. Conclusions

In summary, the dual substitution of Ta for Zr and Mg for Li on the structure and performance of garnet Li_7_La_3_Zr_2_O_12_ is investigated. The newly-developed garnet with a composition of Li_6.5_Mg_0.05_La_3_Zr_1.6_Ta_0.4_O_12_ shows a single cubic phase and exhibits a relatively high total ionic conductivity of 6.1 × 10^−4^ S cm^−1^, which is slightly higher than that of bare Li_6.6_La_3_Zr_1.6_Ta_0.4_O_12_. It may be because the dopant Mg facilitates the densification. Spark plasma sintering is further applied on Li_6.6_La_3_Zr_1.6_Ta_0.4_O_12_ and Li_6.5_Mg_0.05_La_3_Zr_1.6_Ta_0.4_O_12_. The results verify that the SPS process is an effective technology to densify the cubic garnet. Both Li_6.6_La_3_Zr_1.6_Ta_0.4_O_12_ and Li_6.5_Mg_0.05_La_3_Zr_1.6_Ta_0.4_O_12_ show dense microstructures at SPS temperature of 1000 °C. Li_6.6_La_3_Zr_1.6_Ta_0.4_O_12_ possesses the highest room-temperature conductivity of 1.18 × 10^−3^ S cm^−1^ after SPS treatment at 1050 °C, while Li_6.5_Mg_0.05_La_3_Zr_1.6_Ta_0.4_O_12_ shows a conductivity of 8.8 × 10^−4^ S cm^−1^ after SPS procedure at 1000 °C. It is indicated that the relatively high lithium content results in a high ionic conductivity under a similar densification condition.

## Figures and Tables

**Table 1:** Corresponding lattice parameters reported in the literature and the calculated theoretical densities and relative densities of LLZTO.

Compound	Lattice Parameter (Å)	Ref.	Theoretical Density (g cm^−3^)	Relative Density (%)
Li_6.25_La_3_Zr_1.25_Ta_0.75_O_12_	12.91553(6)	[20]	5.561	91.10
Li_6.4_La_3_Zr_1.4_Ta_0.6_O_12_	12.923	[21]	5.551	92.69
Li_6.5_La_3_Zr_1.5_Ta_0.5_O_12_	12.9340	[23]	5.410	90.24
Li_6.6_La_3_Zr_1.6_Ta_0.4_O_12_	12.939	[24]	5.352	94.05
Li_6.7_La_3_Zr_1.7_Ta_0.3_O_12_	12.9721	[21]	5.261	94.48
Li_6.8_La_3_Zr_1.8_Ta_0.2_O_12_	12.9780	[22]	5.203	88.24
